# Evaluating Functional Dispersal in a Nest Ectoparasite and Its Eco-Epidemiological Implications

**DOI:** 10.3389/fvets.2020.570157

**Published:** 2020-10-19

**Authors:** Amalia Rataud, Marlène Dupraz, Céline Toty, Thomas Blanchon, Marion Vittecoq, Rémi Choquet, Karen D. McCoy

**Affiliations:** ^1^MIVEGEC, Univ Montpellier - CNRS – IRD, Centre IRD, Montpellier, France; ^2^CEFE, Univ Montpellier, CNRS, EPHE, IRD, Université Paul Valéry Montpellier 3, Montpellier, France; ^3^Tour de Valat, Research Institute for the Conservation of Mediterranean Wetlands, Arles, France; ^4^Center for Research on the Ecology and Evolution of Disease (CREES), Montpellier, France

**Keywords:** capture-mark-recapture (CMR), multi-state model, vector, tick, Argasidae, colonial seabirds, *Larus michahellis*, *Ornithodoros maritimus*

## Abstract

Functional dispersal (between-site movement, with or without subsequent reproduction) is a key trait acting on the ecological and evolutionary trajectories of a species, with potential cascading effects on other members of the local community. It is often difficult to quantify, and particularly so for small organisms such as parasites. Understanding this life history trait can help us identify the drivers of population dynamics and, in the case of vectors, the circulation of associated infectious agents. In the present study, functional dispersal of the soft tick *Ornithodoros maritimus* was studied at a small scale, within a colony of yellow-legged gulls (*Larus michahellis*). Previous work showed a random distribution of infectious agents in this tick at the within-colony scale, suggesting frequent tick movement among nests. This observation contrasts with the presumed strong endophilic nature described for this tick group. By combining an experimental field study, where both nest success and tick origin were manipulated, with Capture-Mark-Recapture modeling, dispersal rates between nests were estimated taking into account tick capture probability and survival, and considering an effect of tick sex. As expected, tick survival probability was higher in successful nests, where hosts were readily available for the blood meal, than in unsuccessful nests, but capture probability was lower. Dispersal was low overall, regardless of nest state or tick sex, and there was no evidence for tick homing behavior; ticks from foreign nests did not disperse more than ticks in their nest of origin. These results confirm the strong endophilic nature of this tick species, highlighting the importance of life cycle plasticity for adjusting to changes in host availability. However, results also raise questions with respect to the previously described within-colony distribution of infectious agents in ticks, suggesting that tick dispersal either occurs over longer temporal scales and/or that transient host movements outside the breeding period result in vector exposure to a diverse range of infectious agents.

## Introduction

Dispersal is a fundamental process influencing the ecology and evolutionary trajectory of species. It is a major determinant of a species' population dynamics and genetic structure, and as such, conditions the ability of organisms to adapt to new environments (1). True dispersal requires the physical movement of an individual from one patch to another (i.e., functional dispersal), followed by successful reproduction (i.e., effective dispersal). Genetic studies have been extremely useful for measuring effective dispersal, particularly in organisms that are hard to follow directly [e.g., (2)], but these studies can only provide estimates of dispersal rates when genetic structure occurs and cannot inform us about physical movement when post-movement reproduction is not successful. Functional dispersal is nevertheless essential to understand when one wants to predict expansion/invasion dynamics and associated colonization success (1), or when examining disease circulation in cases when the transient presence of an individual is enough for pathogen transmission to occur. However, measuring functional dispersal can be difficult because the ability to follow individual animals depends on their biology and ecology.

Capture-mark-recapture (CMR) studies have contributed much to our understanding of movement and are frequently used to study population dynamics and dispersal of vertebrates (3). These methods are only rarely applied to invertebrates (4–8). Although many studies have successfully marked and released arthropods to determine dispersal distances [e.g., (9, 10)], obtaining sufficient data for subsequent statistical analyses is difficult, limiting our ability to make robust inferences on movement in many groups. The present study focuses on the functional dispersal of the seabird tick, *Ornithodoros maritimus*, a member of the Argasidae or soft tick family, using CMR methodology.

Ticks are among the most important disease vectors worldwide, transmitting a wide variety of infectious agents including bacteria, viruses, and eukaryotic parasites (11) to a multitude of vertebrate hosts including birds, reptiles, and mammals (12). There is a general lack of knowledge on tick biology and population dynamics under natural conditions, and this is particularly true for soft ticks which, because of their more endophilic lifestyle and feeding habits, frequently go undetected in host populations (13). Here, we use *O. maritimus* as a model soft tick species to examine functional dispersal at a small spatial scale, among nests within a breeding colony of its host, in order to better understand its role in local population expansion, genetic structure and the transmission of infectious agents among host individuals.

*Ornithodoros maritimus* is commonly found in seabird breeding colonies in the Mediterranean Sea and eastern North Atlantic Ocean (14–17) and may act as vector to numerous infectious agents including diverse bacteria, protozoans and viruses (18–20). Like most argasid ticks, *O. maritimus* has a nidicolous lifestyle and feeds on the host rapidly (several minutes) in nymphal and adult life stages, usually at night when the host is largely immobile (13). This limited contact with the host should result in low among-colony dispersal, and may have a cascading effect on pathogen spread (21). At the within-colony scale, among nest dispersal should mainly depend on the intrinsic movements of the tick itself, as the seabird hosts are generally territorial during the breeding season. However, active dispersal in endophilous ticks like *O. maritimus* is thought to be limited (13). A need for specific environmental conditions could further induce strong site fidelity and homing behavior to specific microhabitats in these ticks. However, a recent study on the among-nest distribution of infectious agents carried by *O. maritimus* found no spatial structure in their presence in ticks (19). As gulls are territorial during the breeding season and tend to show high nest site fidelity between years (22), all ticks in a nest should be exposed to the same infectious agents. If ticks move independently of their host, but only short distances, neighboring nests should have a higher probability of sharing infectious agents than more distant nests. As these patterns were not found, it was suggested that ticks move among host nests frequently enough to disseminate infectious agents across the colony (19).

Here, we test this hypothesis by characterizing functional dispersal of *O. maritimus* within a colony of yellow-legged-gulls during the breeding period. By integrating an experimental field study with detailed CMR data and a multi-state statistical framework (23, 24), we also test if functional dispersal differs according to host nest success, i.e., whether a lack of chicks in the nest may motivate ticks to move more readily, and tick life stage. We only consider nymphal and adult ticks in our study for two reasons. First, applying CMR methods to larvae in the field could not be done for methodological reasons because larvae are too small to repeatedly mark. Second, larval ticks are more susceptible to environmental conditions (25) and are thus less likely to successfully move independently of the host. Based on our current knowledge, we expected higher among nest tick dispersal in failed nests, higher dispersal of male ticks because of lower blood meal requirements and their quest for sexual partners and, higher dispersal in adults than in nymphal ticks because adults are more resistant to environmental conditions (26). By translocating ticks from nearby nests to focal nests, we also tested for homing behavior, which could illustrate site fidelity in *O. maritimus*.

## Materials and Methods

### Biological System

*Ornithodoros maritimus* is a member of the soft tick (Argasidae) complex *Ornithodoros capensis* sensu lato which is currently composed of eight described species that exploit colonial seabirds in the tropical and sub-tropical areas of the world (15). Like other soft tick species, *O. maritimus* has a polyphasic life cycle composed of three active stages: a single larval stage, several nymphal instars and a sexual adult stage (27). Unlike hard ticks (Ixodidae), these ticks feed rapidly on the host (from several minutes in the nymphal and adult stages to several hours in the larval stage) when the host is resting, usually at night (28). Total time on the host is therefore much shorter in soft ticks compared to hard ticks. Dispersal in these ticks can occur by active movement of the ticks themselves, and/or via their hosts. The latter is the only mechanism for inter-colony dispersal for *O. capensis* s.l. ticks. Within colonies, both passive and active dispersal could occur. As mentioned in the introduction, both are expected to be low because of the nidicolous nature of these ticks (13), the territoriality of the gulls, and the fact that ticks do not exploit hosts during the active periods of the day (21). No quantification of dispersal at either spatial scale currently exists for ticks of this group.

*Ornithodoros maritimus* is known to exploit a wide range of colonial seabird host species including cormorants, terns, and gulls from southern Great Britain to the Mediterranean Sea (16). In the Mediterranean region, this tick often exploits breeding yellow-legged gulls (19). Yellow-legged gulls are the most common and widespread seabird of the western Mediterranean (29) and tend to show high ecological adaptability (30). At adulthood, these birds typically breed in dense colonies, laying 2–3 eggs per year in nests built on the ground or on cliff ledges. During the breeding season, they have limited movements, going from feeding areas to the nest territory (31). Outside breeding, *L. michahellis* remains gregarious, concentrating around ports, harbors, and dumps (31). Because of its longevity, nest site fidelity, and seasonal breeding (22), the presence of this bird in the colony area is highly predictable for nest parasites like *O. maritimus* (32).

Despite the limited time that this tick is in contact with the host during the bloodmeal, the repeated nature of these meals may increase the transmission probability of infectious agents carried by the birds and, as a consequence, their prevalence within local populations (21). Indeed, although few investigations exist to date, ticks of the *O. capensis* complex are known vectors of several infectious agents, such as *Borrelia* spp bacteria responsible for relapsing fever in humans (33) and the Soldado virus which can induce high mortality rates in bird populations and pruritus in humans (18, 20, 34). Numerous infectious agents have also been identified in *O. maritimus* in the focal colony of the present study: bacteria including *Anaplasma* spp, *Bartonella henselae, Borrelia* sp., *Coxiella* sp., *Francisella* sp., and *Rickettsia* spp.; protozoan *Babesia* sp., and a virus closely related to the West Nile virus (19). The pathogenic effect of these infectious agents for birds and humans are largely unknown as of yet [e.g., (35)].

### Study Location

**Figure 1:**
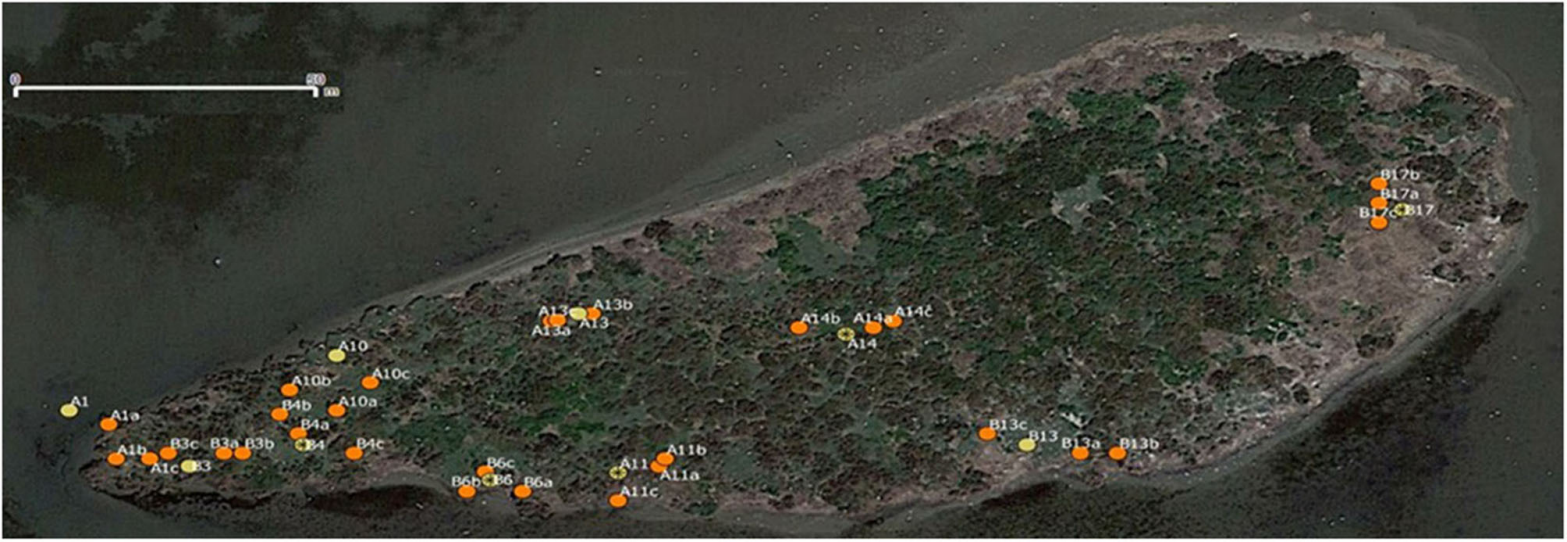
Map showing the position of the 40 tracked nests on Carteau (43°22′39″N 4°51′28″E). Nests are identified according to their status: focal nests (successful = yellow circles, failed = yellow circles with a black star) are labeled with a capital letter followed by a number; peripheral nests (orange circles) are labeled with the name of the focal nest of the same nest group followed by a lower-case letter.

Field work was conducted in the yellow-legged gull colony of Carteau (43°22′39″N 4°51′28″E), a small islet in the Gulf of Fos in the Camargue area of southern France (Figure 1). This flat islet of 1.36 km^2^ (210 m long by 65 m wide) is entirely occupied by breeding yellow-legged-gulls. During the 2018 regional population survey, 275 breeding pairs were counted on Carteau (Tour du Valat, Association des Marais du Vigueirat). *Ornithodoros maritimus* was identified morphologically and genetically from gull nests in past studies and was the only tick species found on Carteau (15, 19).

### Experimental Procedures

Field sampling took place once per week over 5 weeks from April to May 2017. To estimate inter-nest dispersal and the factors that affect it, we selected, marked and recorded the GPS coordinates of 10 nest groups across the islet (Figure 1). Each nest group included four nests: a focal nest and the three closest nests (peripheral nests). The average distance between nests of a group was 6.29 (±3.21) m, whereas the average distance between nest groups was 25.22 (±13.13) m. One half of the focal nests were manipulated for breeding success during egg incubation: five were left in success and five were put in failure (eggs removed). At the time of manipulation, the average clutch size of the studied nests was 2.6. One successful nest failed at the egg stage and one failed nest relaid; the category of these nests was reversed for the analyses. Otherwise all successful nests produced chicks.

**Figure 2:**
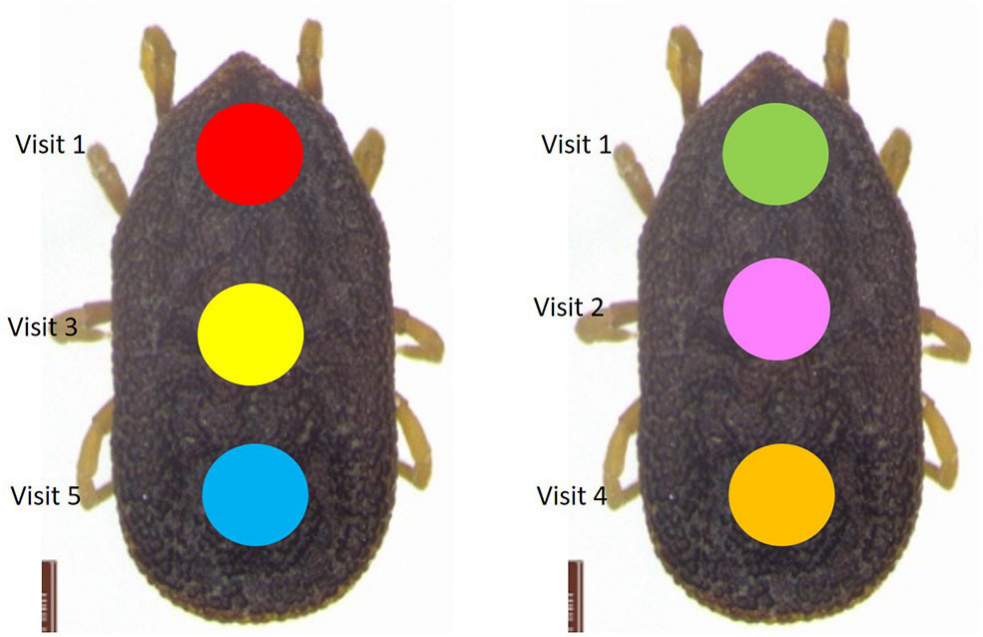
Marking protocol for *Ornithodoros maritimus*. Ticks were initially marked with the color of their nest of origin (visit 1). At each consecutive visit, a date-specific color was used to track capture histories. For example, the individual on the left originated in the peripheral nest B4a and was moved to focal nest B4 during visit 1 (red mark corresponding to the nest of origin B4a). It was then found in focal nest B4 in visits 3 (date-specific yellow mark) and 5 (date-specific blue mark). It was not seen during visits 2 and 4. Its recapture history was thus 10101 (see Supplementary Material). The individual on the right was found in focal nest A1 during visit 1 (green mark corresponding to the nest of origin A1), then again in the same nest in visits 2 (date-specific pink mark) and 4 (date-specific orange mark). In visit 5, it was found in peripheral nest A1a and was collected. It was not found in visit 3. Its recapture history was thus 11012 (see Supplementary Material).

At each field visit, each nest was searched for 3 min by two people (6 min total search time per nest); one person examined the upper nest materials in a white tray while the other searched directly inside the nest. Thirty adult and nymphal ticks from the focal nests and 30 adult and nymphal ticks from the peripheral nests were marked with a spot of acrylic paint (Figure 2) at the first sampling occasion. To test for homing behavior, the 30 ticks from the peripheral nests were placed in the focal nest, such that a minimum of 60 ticks were present in each focal nest. Based on previous studies, this number corresponds to natural infestation levels in moderate to highly infested nests (17, 19). An individual color was attributed to each focal nest and a different color to the three peripheral nests of the same group (20 colors overall). During subsequent sampling occasions, all ticks found during the timed searches were counted, but only the initially marked cohort was followed in detail. At each visit, these marked ticks received a date-specific color to indicate their recapture history (Figure 2). The life stage and sex of the ticks were recorded at each visit. Any ticks that dispersed to the peripheral nests were collected.

### CMR Modeling

To estimate dispersal rates of *O. maritimus* within the colony, we applied a multistate CMR model to the dataset (36). CMR modeling is based on individually marking part of a population. Marked individuals are followed over time during several recapture occasions. The recapture history of an individual is composed of a succession of detection and non-detection events, respectively, noted 1 and 0. For example, 10100 indicates that the individual was detected on the first and third occasions, but not on the second, fourth, and fifth. CMR modeling has the particularity of taking the probability of detection into account in order to obtain unbiased demographic estimates (survival, dispersal). “Events” code the observations made at time *t* (i.e., detection or not during the sampling occasion), whereas “states” define physiological or geographical states (i.e., individual alive or dead) between time *t* and *t*+*1*. In this study, encounter histories were coded with 4 events. For each recapture occasion, ticks were either not observed (coded 0), observed in a focal nest (coded 1), observed in a peripheral nest (coded 2), or found dead (coded 3). Thus, events were: {not observed (0), observed in site 1 (1), observed in site 2 (2), found dead (3)}. Moreover, four states were defined to describe the data. Indeed, ticks could be present in the focal nests (noted site 1), present in the peripheral nests (noted site 2), just dead (since the last weekly visit, noted J^†^) or dead (over a week, noted†). We assumed “just dead” individuals were ticks found dead in the nest, whereas “dead” individuals were ticks that were no longer capturable (because they died some time ago). As no tick was found dead in site 2, we did not need to specify the site for the state “dead.” States were thus: {site 1, site 2, J^†^, †}. The multistate model is described in more detail in the Supplementary Materials.

As individuals could differ according to characteristics like life stage or sex, the effect of these covariates on demographic parameters were directly included in the model sets (see below). Model selection was performed using AIC values corrected for sample size (QAICc), with the best fit model providing information on the relative influence of different included factors.

### Model Set

#### Model 1: Tick Life Stage

First, we tested whether survival (S), detection probability (P) and inter-nest dispersal (Ψ) varied in relation to tick life stage. In the null model, survival and detection were coded as being constant across tick stages and nest success; these variables were then added in alternative models. No effect of tick origin (tick from focal or peripheral nest) was expected on these two parameters and this factor was therefore not included in the model set. We modeled dispersal in relation to tick stage, origin and nest success.

#### Model 2: Tick Sex

We then tested if survival (S), detection probability (P), and inter-nest dispersal (Ψ) varied in relation to adult tick sex. In the null model, survival and detection were again coded as constant across sexes and nest success, and then added to alternative models. Likewise, no effect of tick origin (tick from focal or peripheral nest) was expected on these two parameters and this factor was therefore not included in the model set. We modeled dispersal in relation to adult tick sex, origin, and nest success.

Model selection and parameter estimation were performed using Program E-SURGE 1.8 (37, 38). The selected model in each model set had the smallest QAICc and two models were deemed to be equivalent when they differed by <2 (39).

## Results

### Tick Sampling

At the first field visit, 578 ticks (189 adult males, 249 adult females, and 140 nymphs) were marked. In total, 138 ticks (30 adult males, 77 adult females, and 31 nymphs) were recaptured at least once, representing 23.9% of the initial number. Three ticks were found dead in focal nests and nine were recaptured in peripheral nests and collected.

### Model Selection

#### Model 1: Tick Life Stage

The QAICc values of the different models were all very close for model set 1, and no one model was selected. However, the models with smallest QAICc suggested an effect of tick origin and nest success on dispersal (Table 1); no effect of tick life stage was evident. As no one model best described the data, we did not attempt to estimate demographic parameters for this analysis.

**Table 1:** Model selection results for model 1, taking into account different life stages of *Ornithodoros maritimus*.

**Model**	**Number of parameters**	**Deviance**	**QAIC**	**QAICc**
S_cst_ P_cst_ Ψ_nestsuccess.tickorigin_	7	1,109.5988	1,123.5988	1,123.7529
S_nestsuccess_P_nestsuccess_Ψ_nestsuccess.tickorigin_	9	1,105.8826	1,123.8826	1,124.1309
S_nestsuccess_P_cst_Ψ_nestsuccess.tickorigin_	8	1,108.0607	1,124.0607	1,124.2591
S_cst_P_tickstage_Ψ_nestsuccess.tickorigin_	8	1,109.0804	1,125.0804	1,125.2787
S_tickstage_P_cst_Ψ_nestsuccess.tickorigin_	8	1,109.5364	1,125.5364	1,125.7347

*Survival (S) and detection probability (P) were modeled as constant (cst) and depending on tick stage and nest success. Dispersal was modeled as a constant (cst), and depending on tick stage, nest success and tick origin. Only the top five of 128 models are presented. The number of parameters and the deviance were used to calculate QAICc (Akaike Criterion corrected for sample size) of each model. The selected model has the smallest QAICc. The complete model set is available at https://doi.org/10.5281/zenodo.2591254*.

#### Model 2: Tick Sex

The selected model from the model 2 set revealed a difference in tick survival according to nest success and an effect of sex and nest success on the detection probability. There was also a difference in tick dispersal according to origin and nest success, but not according to tick sex (Table 2).

**Table 2:** Model selection results for model 2 that considers only adult *Ornithodoros maritimus*.

**Model**	**Number of parameters**	**Deviance**	**QAIC**	**QAICc**
S_nestsuccess_ P_ticksex.nestsuccess_ Ψ_nestsuccess.tickorigin_	11	844.0884	866.0884	866.5692
S_nestsuccess_ P_ticksex.nestsuccess_ Ψ_nestsuccess_	9	850.2589	868.2589	868.5856
S_ticksex.nestsuccess_ P_nestsuccess_ Ψ_nestsuccess.tickorigin_	11	847.1897	869.1897	869.6706
S_nestsuccess_ P_ticksex_Ψ_nestsuccess.tickorigin_	9	852.1825	870.1825	870.5092
S_tickstage.nestsuccess_ P_nestsuccess_ Ψ_nestsuccess_	9	853.3609	871.3609	871.6876

*Survival (S) and detection probability (P) were modeled as constant (cst) and depending on tick sex and nest success. Dispersal was modeled as a constant (cst), and depending on tick sex, nest success and tick origin. Only the top five of 128 models are presented. Model selection was performed ass outlined in Table 1. The complete model set is available at https://doi.org/10.5281/zenodo.2591254*.

### Estimated Parameters From Model 2

The survival probability of *O. maritimus* differed according to nest success. As expected, the one week survival probability of ticks in successful nests [0.609, IC_95%_ = (0.495; 0.712)] was higher than that in failed nests [0.381, IC_95%_ = (0.295; 0.475)].

**Figure 3:**
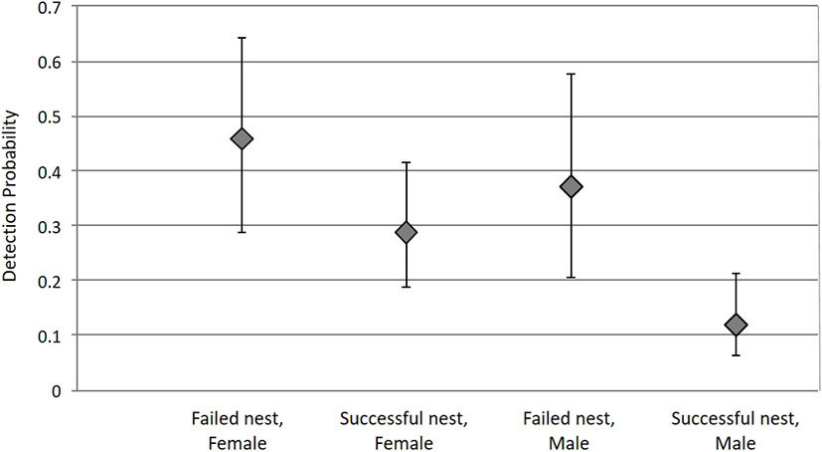
Tick detection probability depends on the interaction between nest success (failed or successful) and tick sex (female or male). Bars represent 95% confidence intervals.

The detection probability varied with tick sex and nest success. Detection of females in failed nests was higher than that of females in successful nests [females, failed = 0.459 [IC_95%_ = (0.286; 0.642)]; females, successful = 0.289 (IC_95%_ = [0.189; 0.414])]. Detection of males was lower in general, but followed the same trend in relation to nest success (males, failed = 0.37 (IC_95%_ = [0.207; 0.575]) and males, successful = 0.119 (IC_95%_ = [0.063; 0.214]); Figure 3).

**Figure 4:**
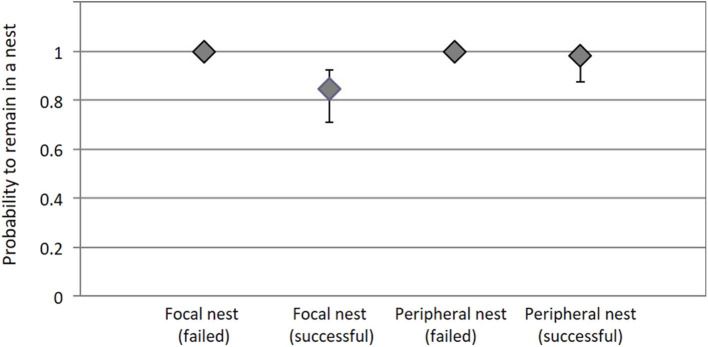
The probability that a tick remains at a nest site (Ψ_11_) depends on the interaction between nest success (failed or successful) and tick origin (focal or peripheral nest). Bars represent 95% confidence intervals.

Overall, inter-nest dispersal rates of ticks were very low, but some dispersal did occur. Surprisingly, ticks in successful nests tended to disperse more than ticks in failed nests. Moreover, in successful nests, ticks from focal nests tended to disperse more than ticks from peripheral nests. The probability that ticks were present and alive on a site at time *t* and present and alive on the same site at time *t*+*1* was 1,00 (IC_95%_ = not available) in focal and peripheral nests in failure, 0.846 (IC_95%_ = [0.709; 0.925]) in focal nests in success and 0.980 (IC_95%_ = [0.873; 0.998]) in peripheral nests in success (Figure 4).

## Discussion

In this study, we characterize functional dispersal in the soft tick *O. maritimus* at a small spatial scale, among nests within a colony of yellow-legged gulls, using capture-mark-recapture data. Estimated inter-nest dispersal rates of ticks were very low overall, indicating a low tendency for *O. maritimus* to move among nests and confirming a strongly endophilous lifestyle in this tick species.

Few studies to date have attempted to measure arthropod movements using CMR modeling, largely due to the difficulty in marking and recapturing individuals. Here, we focused on a short time period when ticks could be followed with a low probability of losing their marks (over a few weeks during the host breeding season). Given the relatively large size of the ticks (about 4 mm; 11), we were also able to place multiple marks that enabled us to directly follow the capture history of each individual. Using this data, we observed a recapture rate of 24%. This rate is relatively high for an arthropod model (5), providing us with enough data to estimate demographic parameters. Other studies that marked ticks found similar recapture rates in adult female ticks, including in one soft tick, *Ornithodoros moubata* (25%) (4, 7, 8, 10)], suggesting that this approach may work well for these arthropods. According to the selected model from the model 2 analysis, which considered only adult ticks, the detection probability of *O. maritimus* seems to depend on tick sex; there was a significantly lower detection probability for male ticks (0.245 for males compared to 0.374 for females). This difference could either be due to the smaller body size of male ticks, or to sex-specific behavioral differences. In the latter case, making predictions about behavior and detection are not obvious because there are several reasons that time spent in the nest may differ between the sexes, depending, for example, on where females prefer to lay eggs and where copulation takes place; these elements are unknown for *O. maritimus*. Surprisingly, the detection probability of soft ticks also seemed to depend on nest success, with higher detection in failed nests. However, again this may be due to behavior, where engorged ticks leave the nest area to molt or lay eggs. Detailed behavioral studies are now required to test these hypotheses.

As the top ranking models did not include an effect of tick life stage (see model 1 results), we did not estimate detection probabilities for nymphal ticks. Although one of the five top models suggested a potential effect of life stage on detection, the observed proportion of global recaptures for nymphs (22.1%) and adults (24.4%) was similar. This suggests that detection may not differ strongly between two life stages and mark loss due to nymphal molts may not occur within the studied period. However, the overall lower proportion of followed nymphs (~24%) compared to adults (~76%) could have lowered our ability to detect an effect.

Neither tick life stage nor tick sex was found to impact survival probability. However, survival probability of *O. maritimus* did differ according to nest success and was higher in nests when chicks were present. This was expected as the ability to have ready access to a host for the bloodmeal should improve tick fitness. Indeed, the quality of the bloodmeal is known to influence the success and duration of the life cycle in argasid ticks (13). However, the survival probability of *O. maritimus* does not seem to depend only on feeding ability, as it was still estimated at 38% in failed nests. Nest-associated parasites often have to survive long periods without hosts, and those parasites associated with pelagic seabirds may represent an extreme [e.g., (40)]. Indeed, colonial seabirds are frequently only present for a few months per year at the nest site, during the breeding season. The rest of the time, they can wander over vast zones and remain largely (or completely) at sea and are therefore unavailable for exploitation (32). In such cases, dormancy behavior becomes essential for parasite survival, allowing them to wait, sometimes under extreme environmental conditions, until the host is available again. *Ornithodoros* ticks are known to survive long periods (years) without a host if microclimatic conditions are appropriate (13). We therefore feel that our survival estimates are robust. However, one could also postulate that these estimates are distorted by the presence of transient ticks (41), individuals that are considered dead, but which were simply unrecapturable because they permanently emigrated outside the studied area. Analyses realized on data collected in 2018 have shown that this hypothesis does not have high support (42).

In contrast to predictions based on the distribution of infectious agents in ticks within the colony (19), overall inter-nest dispersal rates of *O. maritimus* were very low. However, tick dispersal depended on their origin (focal or peripheral nest), with ticks from focal nests tending to disperse more than ticks from peripheral nests. This was unexpected, and particularly so if ticks have a homing response, i.e., a preference to return to a specific, known microhabitat. Ticks displaced from peripheral to focal nests could have had less energy to allocate to dispersal than local ticks because of the energetic costs of acclimating to another nest environment or having access to fewer bloodmeals post-dispersal. From our results, there is absolutely no indication of homing behavior in displaced ticks.

We expected the dispersal of *O. maritimus* to depend on nest success, with ticks in failed nests dispersing more than ticks in successful nests. Contrary to this prediction, dispersal of soft ticks does not seem to be induced by the quest for a bloodmeal. This may reflect the ability of these ticks to survive long periods of time without a host (13) and highlights the importance of a flexible dormancy strategy where quiescence can offset the costs of limited dispersal in endophilous species. We also found that ticks in successful nests dispersed more than ticks in failed nests. This could again be because ticks in failed nests, unable to feed, may lack enough energy to move.

Here, we examine active tick movement, but dispersal of *O. maritimus* via host movement is of course possible. Given the short duration and timing of the tick bloodmeal and the limited movements of yellow-legged gulls within the colony during the breeding period (43), we considered this unlikely. Indeed, no effect of chick presence on the dispersal of *O. maritimus* was indicated in analyses from 2018, tick movement did not increase at the time that chicks started to move around the colony (42). However, the role of host movements in tick dispersal later in the year and at different spatial scales remains unknown. A population genetic study of ticks at the among-nest scale could shed light on the role of the host in local dispersal events.

We also expected dispersal in *O. maritimus* to depend on tick sex, with higher dispersal in male ticks, due to their reduced need for bloodmeals and their quest for sexual partners. Although past studies have documented male-biased dispersal in ticks (44), we found no support for this. Future population genetic analyses would also enable us to test the hypothesis of sex-biased dispersal in *O. maritimus*.

We found that *O. maritimus* has low functional dispersal rates among nests within the host breeding colony. This result is consistent with the general idea that soft ticks tend to be strongly endophilous (13). The sedentary lifestyle of *O. maritimus* should restrict gene flow among natural populations resulting in high phenotypic variability and genetic structure among populations (13, 21). A lack of gene flow could mean a limited role of this soft tick in the circulation of associated infectious agents. Although transmission may occur more readily in soft tick systems compared to hard tick systems, because soft ticks repeatedly feed in nymphal and adult life stages (21), without dispersal an infected tick can only transmit its infectious agents to hosts breeding in the same nest site (i.e., family members). Again, a genetic approach examining dispersal could help us determine the role of tick dispersal in the transmission of associated infectious agents, particularly for larval ticks which feed for longer periods of time on the host compared to nymphal and adult stages (several hours compared to several minutes). It would also allow us to infer whether seabird presence in the colony outside the breeding season could result in exposure of active ticks to novel host individuals, potentially explaining observed patterns in pathogen prevalence in ticks (19).

## Conclusions

Knowledge on functional dispersal, describing physical movements of individuals from one patch to another, is essential to understand population dynamics and to predict ecological and evolutionary changes in a species. Functional dispersal can be particularly important to take into account in the case of vectors like ticks, because these ectoparasites affect host reproduction and can transmit infectious agents. Our capture-mark-recapture (CMR) study has allowed us to identify some of the factors influencing inter-nest dispersal probability of the soft tick *O. maritimus* at a small spatial scale, within a colony of yellow-legged gulls, taking into account both tick survival and detection probability. These first results have highlighted a weak dispersal propensity in this tick and suggest a limited role of active tick movement in the circulation of associated infectious agents at the within-colony level. Although survival and inter-nest dispersal of *O. maritimus* seem to depend on nest success (host availability), analyses did not indicate homing behavior. The detection probability of *O. maritimus* also depended on nest success and tick sex, but not in the predicted directions. More in-depth knowledge on the biology of this tick is now required to fully interpret these results and should prove useful for future work on this biological system. Although the present study represents one of the first applications of CMR modeling to an arthropod system using multiple recapture events, more information on tick dispersal at larger spatial and temporal scales is now necessary to better understand its population dynamics, the potential impact of these dynamics for the seabird host, and the circulation of infectious agents within the Mediterranean Basin.

## Data Availability Statement

The datasets presented in this study can be found in online repositories: https://doi.org/10.5281/zenodo.2591254.

## Ethics Statement

Ethical review and approval was not required for the animal study because the only manipulation presented in the study involved the modification of gull reproductive success-i.e., egg removal. This species is under control programs and approval for this manipulation of reproductive success was given by the local autorities (Grand Port Maritime de Marseille and DDTM 13/Service Mer Eau Environnement/Pôle Nature et Territoires n°13-2018-02-2-003).

## Author Contributions

KM, RC, and MV designed the initial study. KM, MV, TB, CT, and MD contributed to the practical implementation of the design and carried out the field work. AR and RC organized the data and performed the CMR analyses. All authors contributed to data interpretation. AR, RC and KM wrote the first draft of the manuscript and all authors contributed to revisions.

## Conflict of Interest

The authors declare that the research was conducted in the absence of any commercial or financial relationships that could be construed as a potential conflict of interest. The handling Editor declared a past co-authorship with the authors KM and CT.
