# Supplementary material for: Evaluating Functional Dispersal in a Nest Ectoparasite and Its Eco-Epidemiological Implications
Source: Front Vet Sci. 2020 Oct 19;7:570157. doi: 10.3389/fvets.2020.570157 (PMC7604267; doi:10.3389/fvets.2020.570157)
Supplement: Supplementary file 1 [file Data_Sheet_1.docx]

**Supplementary Materials**

In this study, encounter histories were coded with 4 events at each recapture occasion {not observed (0), observed in site 1 (1), observed in site 2 (2), found dead (3)}. Indeed, the state of ticks could be present in the focal nests (noted site 1), present in the peripheral nests (noted site 2), just dead (since the previous week, noted J†) or dead (for over a week, noted †). Multistate models permit one to estimate the encounter probability P (the probability that an individual is encountered at a site *M* at time *t* knowing that it is alive and present at this site *M* and time *t*), the survival probability S (the probability that an individual alive at a site *M* at time *t* is still alive at time *t+1*) and the movement probability Ψ (the probability that an individual disperses from a site *M* at time *t* to site *N* at time *t+1*) (Lebreton, 2009). Survival and movement conditional on survival are considered as two different steps in transition matrices (Lebreton, 2009).

Matrix patterns for initial states describe the states at the first occasion (marking) when all ticks are in the focal nest, noted: Π = {1, 0, 0} for the proportion of individuals on site 1, site 2 and dead, respectively. The encounter matrix describes the recapture and the recovery process. For example, p1 indicates the probability that the individual is detected in site 1 (focal nests) given that it is present in site 1 and 1-p1 indicates the probability that an individual is not detected given that it is present in site 1. The recovery probability 𝝀 is the probability that individuals that just died were recaptured; we assumed that no individual dead for over a week was recovered:

Model selection was performed using program E-SURGE 1.8 (Choquet and Nogue, 2011) with an Akaike Information Criterion corrected for sample size (QAICc; Akaike, 1973). 128 models were considered (see model sets 1 and 2 : <https://doi.org/10.5281/zenodo.2591254>). The selected model had the smallest QAICc value, and two models were considered different when their QAICc differed by more than two.
